# Changes in Nasal Anatomy and Airflow After Endoscopic Resection of Pituitary Adenomas Using Computational Fluid Dynamics: A Pilot Study

**DOI:** 10.7759/cureus.85568

**Published:** 2025-06-08

**Authors:** Filip Trnka, Hana Schmirlerova, Michal Schmirler, David Netuka, Kristýna Sichová, Martin Cerný, Thomas Hummel, Martin Majovský

**Affiliations:** 1 Faculty of Mechanical Engineering, Department of Fluid Dynamics and Thermodynamics, Czech Technical University in Prague, Prague, CZE; 2 First Faculty of Medicine, Department of Neurosurgery and Neurooncology, Charles University and Military University Hospital, Prague, CZE; 3 Department of Otorhinolaryngology, University of Dresden Medical School, Dresden, DEU

**Keywords:** adenomas, computational fluid dynamics (cfd), computed tomography (ct), nasal cavity, pituitary tumour

## Abstract

Purpose: Pituitary adenoma, a relatively common intracranial tumor, is often treated surgically through the nasal cavity, which alters its anatomy. This study aims to determine the severity of these changes in airflow and flow distribution within the nasal cavity, focusing on the anterior nasal region's role in airflow redistribution. Computational fluid dynamics (CFD) was employed to analyze these changes before and after surgery.

Methods: Data from four patients of the Department of Neurosurgery and Neuro-oncology of the Military University Hospital, Prague, were analyzed using CFD simulations in Ansys Fluent 2021 R1. Computed tomography (CT) scans were used to model the nasal cavities pre- and post-surgery, creating polyhedral meshes of 1.8 million cells before surgery and 2.2 million cells after surgery. The k-ε turbulent model was applied to compute flow fields, providing consistent results across patients.

Results: The surgery increased the nasal cavity volume, primarily due to the endonasal transsphenoidal approach. Cross-sectional areas, particularly in the middle nasal meatus, were enlarged, reducing airflow velocity without altering total volume flow. Most airflow was redistributed through the middle nasal meatus, while flow in peripheral regions decreased. The anterior part of the nasal cavity was identified as having the most significant influence on airflow redistribution.

Conclusion: Surgery impacts nasal anatomy and airflow dynamics significantly, particularly in the anterior part of the nasal cavity. These findings emphasize the need for surgical precision to minimize unintended shifts in airflow patterns. Further studies are recommended to validate these observations.

## Introduction

Endoscopic pituitary surgery is a modern field of neurosurgery that uses an endoscopic technique to resect pituitary tumours, mainly pituitary adenomas. The endoscope is inserted into a nostril, and the pituitary adenoma is approached through the nasal cavity and the sphenoid sinus. A surgical route through the nasal cavity is minimally invasive in the sense that the technique avoids the traditional skull opening, requiring the creation of a bone flap (craniotomy) and causing head scarring, among other possible complications. However, the endoscopic endonasal approach has potential complications, especially postoperative nasal morbidity (e.g., olfactory dysfunction, nasal crusting, synechiae, and chronic sinusitis) [1].

One of the most severe nasal complications of pituitary surgery is the complete (anosmia) or partial (hyposmia) loss of olfaction, occurring in approximately 3% of patients [2]. Olfaction is an important sensory input that affects several activities, including detecting hazards and food consumption. Olfactory dysfunction negatively impacts quality of life (QoL), especially in terms of food intake, safety, personal hygiene, and sexual function [3]. Studies have shown that individuals with olfactory dysfunction often have symptoms of depression [4,5]. 

During standard endoscopic endonasal pituitary surgery, the olfactory mucosa on the roof of the nasal cavity should not be directly contacted. However, instruments and endoscopes are repeatedly inserted and removed from the nasal cavity, and some damage to the nasal mucosa is almost inevitable. Such damage could cause postoperative synechiae and possibly block the olfactory cleft. Other anatomical changes in the nasal cavity after surgery may include a deviation of the nasal septum, perforation of the posterior nasal septum, and lateral displacement of the middle turbinate [1,2].

Even subtle anatomical alterations can affect nasal airflow, causing air to bypass the olfactory mucosa and resulting in olfactory dysfunction [6,7,8]. Simulation of nasal airflow in altered nasal anatomy provides a realistic depiction of the effects of surgery on nasal physiology. Therefore, we implemented computational fluid dynamics (CFD) to analyze these airflow changes, with the aim of eventually modifying surgical techniques to improve patient outcomes. CFD is a powerful tool for modelling fluid flow motions and is often used in the nasal airflow simulation. For example, cases of airflow into the nasal cavities with septal perforations in different parts of the nasal cavity were successfully simulated. To the best of our knowledge, nasal airflow after endoscopic resection of the pituitary adenoma using CFD modelling has not yet been studied [9-13].

In this study, our objective was to use CFD to examine changes in nasal airflow before and after endoscopic pituitary surgery, particularly focusing on the olfactory mucosa. We hypothesize that endoscopic pituitary surgery results in significant changes in nasal airflow patterns, particularly affecting the olfactory mucosa, which may contribute to postoperative olfactory dysfunction [14]. Our pilot study aimed to provide a framework for understanding these changes and formulate strategies to mitigate negative outcomes.

## Materials and methods

Process of making models

We enrolled four male patients with pituitary adenoma who underwent endoscopic endonasal surgery in the Department of Neurosurgery and Neuro-oncology of the Military University Hospital in March 2019. Surgery was performed under general anaesthesia using a four-hand bi-nostril technique. The left nostril serves as the main surgical corridor, where an endoscope and one to two instruments are inserted. To gain more working space, the left middle turbinate is pushed laterally. The right nostril is used after posterior septectomy is performed and is used for inserting a secondary instrument. Before and six months after surgery, each patient had two sets of DICOM-formatted, anonymous computed tomography (CT) scans. Slicer 3D software was used to process CT images of all four patients before and after surgery.

**Figure 1:**
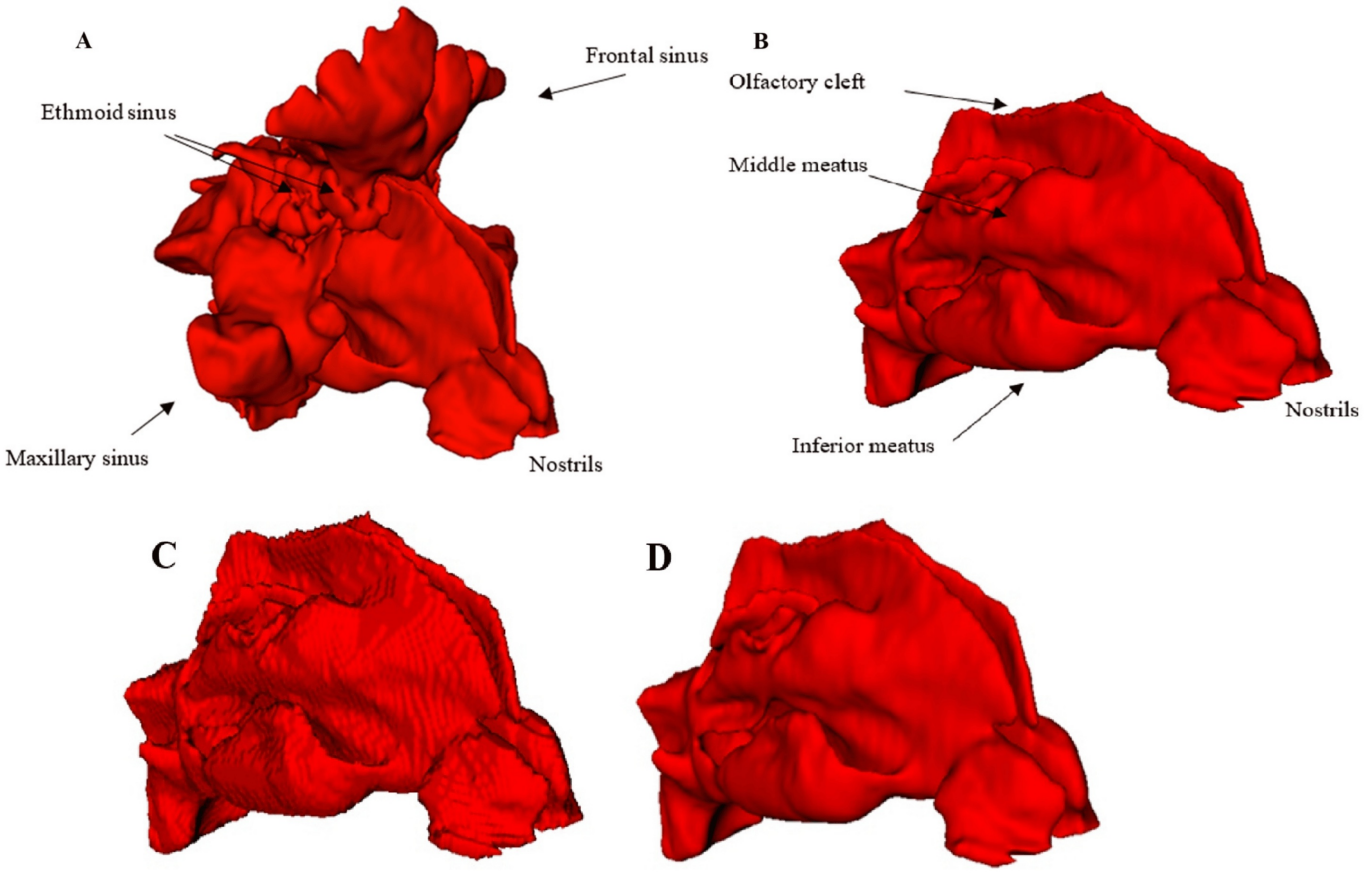
A) Model of the nasal cavity with secondary sinus from the slicer using the threshold function. B) Model of the nasal cavity after erasing secondary cavities. C) Comparison of smoothing function in Slicer model of nasal cavity without smoothing and D) Comparison of smoothing function in Slicer model of nasal cavity with 0,3 Gaussian smoothing. Author's own creation

Models were created based on CT data from patients in DICOM format. The part of the CT scan areas containing the nasal cavity was selected using a threshold function. This function was used to select areas of the scans based on a grayscale. With patients, it was necessary to choose areas without tissue, such as the sinuses. The final model with secondary sinuses is presented in Figure 1A. The entire process of creating a model is described in [15].

For the purpose of modelling flow with widely used CFD software, the sinuses are removed due to their lower influence on flow through the nasal cavity, as described in Mylavarapu et al.'s and Cakmak et al.'s studies [16,17]. Because the areas selected by the threshold function contain secondary sinuses in addition to the nasal cavities, the erase, scissors, and islands functions are used in the next step of the model development procedure. These functions remove parts that are not directly connected to the nasal cavity from the model.

Processing of CT scan data can lead to anatomical misinterpretations at some points in the model. Therefore, after removing parts of the model using erase functions, the models must be checked for the anatomical structure of the nasal cavities. Figure 1B presents the model after the secondary sinuses have been removed.

The models before and after pituitary tumour surgery should be compared to the same coordinate systems in Slicer. Setting the same coordinate system greatly simplifies the comparison of the results in the post-procedure view, especially if the geometry of the nasal cavities changes due to surgery [15].

The distance between the individual CT source images greatly influences the accuracy of the sinus model. The shorter the distance between the source images, the more accurate the models will be. For models with larger CT scan spacings, it is necessary to smooth their surface to correct for a steplike surface. The 3D Slicer programme uses several functions to smooth the model; the Gaussian smoothing function, based on the standard deviation of the Gaussian kernel, was used for the models in this article. Figure 1C-1D illustrates the differences between the smoothing surface and the model without the smoothed surface [18].

**Figure 2:**
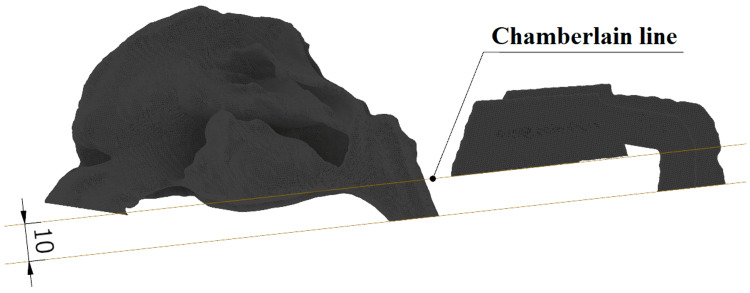
Chamberlain line and the creation of a parallel plane in the nasopharynx region. Author's own creation

In the Slicer, all surfaces of the sinus model are rounded when employing Gaussian smoothing. This is undesirable for the surfaces of the nostrils and nasopharynx. To reproduce planar surfaces at the model's start and end, the model is then updated in CAD software. The entrance length is then created using the nasopharyngeal surface. The entrance lengths of the model are intended to meet the conditions of fluid mechanics theory to produce a laminar (or turbulent) velocity profile. To retain the consistency of the models, they are placed in the nasopharyngeal area parallel to the Chamberlain line as displayed in Figure 2 [15].

The aligned planes created in the CAD software are further set in Space Claim for later selection of boundary conditions for the computational model. The nostril surfaces will be designated as inlet surfaces, and the surface at the end of the entrance length will be set as the outlet. In addition, correction functions are applied in Space Claim to improve the result of subsequent meshing [15].

Meshing

The Ansys Fluent 2021 R1 software was used to mesh all patient models without using the serial meshing mode. The same meshing parameters were set for all models. These parameters were selected based on a mesh study presented in the master thesis of the first author, in which the results of models with different cell counts and mesh parameters were compared to ensure the independence of the solution.

The minimum size of the surface mesh was 0.5 mm, and the maximum cell sizes were set at 2.5 mm. Polyhedral cells were used to create volume meshes because the flow field in the nasal cavity changes dramatically with geometry. The volume mesh cell size was set to 2.5 mm with the addition of creating five prismatic layers. These settings and procedures enabled the creation of a volumetric mesh with orthogonal quality better than 0.15.

**Figure 3:**
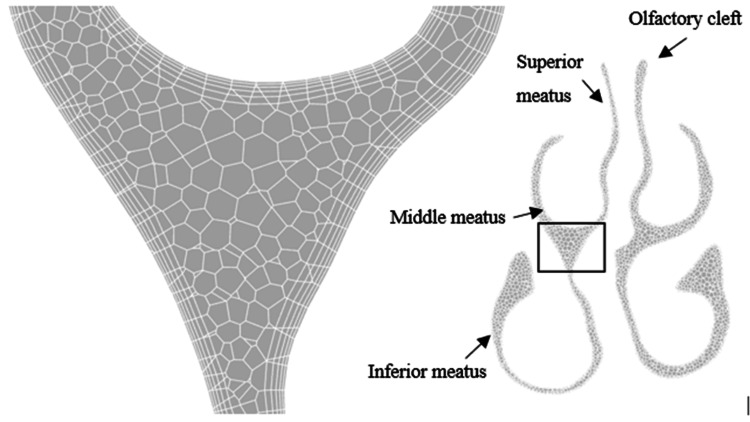
Cross-section of the volume mesh with details of the prismatic layers and description of the anatomical zones of a patient with septal deviation to the right. Author's own creation

Volume mesh models were made for the four patients before and after adenoma surgery. There were approximately 1.8 million cells in the meshes before and 2.2 million after surgery. The increased volume of the sinuses accounts for more cells in the model after surgery. The endoscope and other surgical tools were inserted into the nasal cavity during the removal of an adenoma, increasing the capacity of the cavities. The volume mesh with prismatic layers is shown in Figure 3.

Boundary condition

The created planes in front of the nostrils are set as an inlet in Fluent. The boundary condition of the pressure inlet is placed on the surfaces in front of the nostrils. The boundary condition for the mass flow output is then specified to apply to the planes at the end of the entrance length. The mass flow rate values through the cavities are derived from the human breathing cycle, which has been modified for use in CFD simulations, as described in [19].

**Figure 4:**
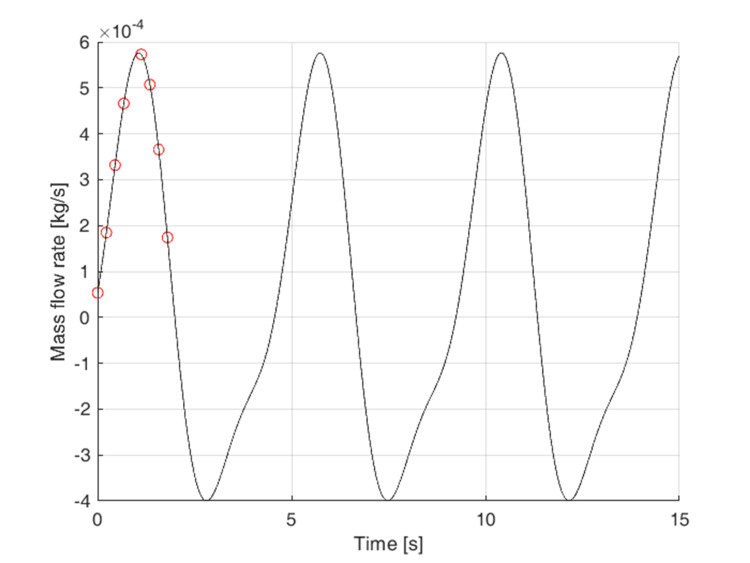
Approximation of the respiratory cycle. The red circles indicate the mass flow values employed in the calculation during inspiration. Author's own creation

With regard to olfactory functioning, inhalation is the most significant phase of the breath cycle. Figure 4 shows the general progression of the respiratory cycle. The red circles indicate the mass flow values employed in the calculation during inspiration. Because the difference in pressure loss between steady and unsteady computational cases is negligible at maximum values of the mass flow rate, as shown in Mösges' study [20], only the maximum mass flow values were used for all calculations in this study.

Solution

The numerical study was performed under steady airflow conditions. Reynolds numbers (Re) in the nostrils ranged from 570 to 1980. Although this range could suggest the use of a laminar model, the local Re in the narrow parts of the middle nasal cavities exceeded the critical Re number. Furthermore, the geometric complexity of the nasal cavity required the use of a turbulent model for accurate calculations. Similar approaches using turbulent models have been described and used in studies [21-25], which highlight the importance of capturing complex flow patterns and using turbulent models for calculations of flow characteristics in the nasal cavity.

Given the potential for anisotropic flow due to the intricate geometry of the nasal cavity, the realisable k-ε turbulent model was selected based on our experience and its proven effectiveness in handling such conditions. The values of y+ and other significant variables were computed from the initial simulations to assess the quality of the model. Since the value of y+ was less than 5 in all models, the enhanced wall treatment function was used to accurately resolve the flow in the prismatic layer region.

Patients and ethics statement

This study was carried out on CT data from four patients before and after surgery. Table 1 displays information about each of the patients’ pathologies.

**Table 1:** Patients' information.

ID	Sex	Age	Anatomical abnormalities
1	Male	58	Septal deviation to right both before and after surgery
2	Male	60	No nasal pathology
3	Male	60	Septum perforation both before and after surgery
4	Male	43	Synechia after surgery

The study was conducted in accordance with the principles of the Declaration of Helsinki. Ethical approval was obtained from the Ethical Committee of the Military University Hospital Prague (no. 108/15-4/2020), and informed consent was acquired from all participants prior to inclusion in the study.

Post-processing

**Figure 5:**
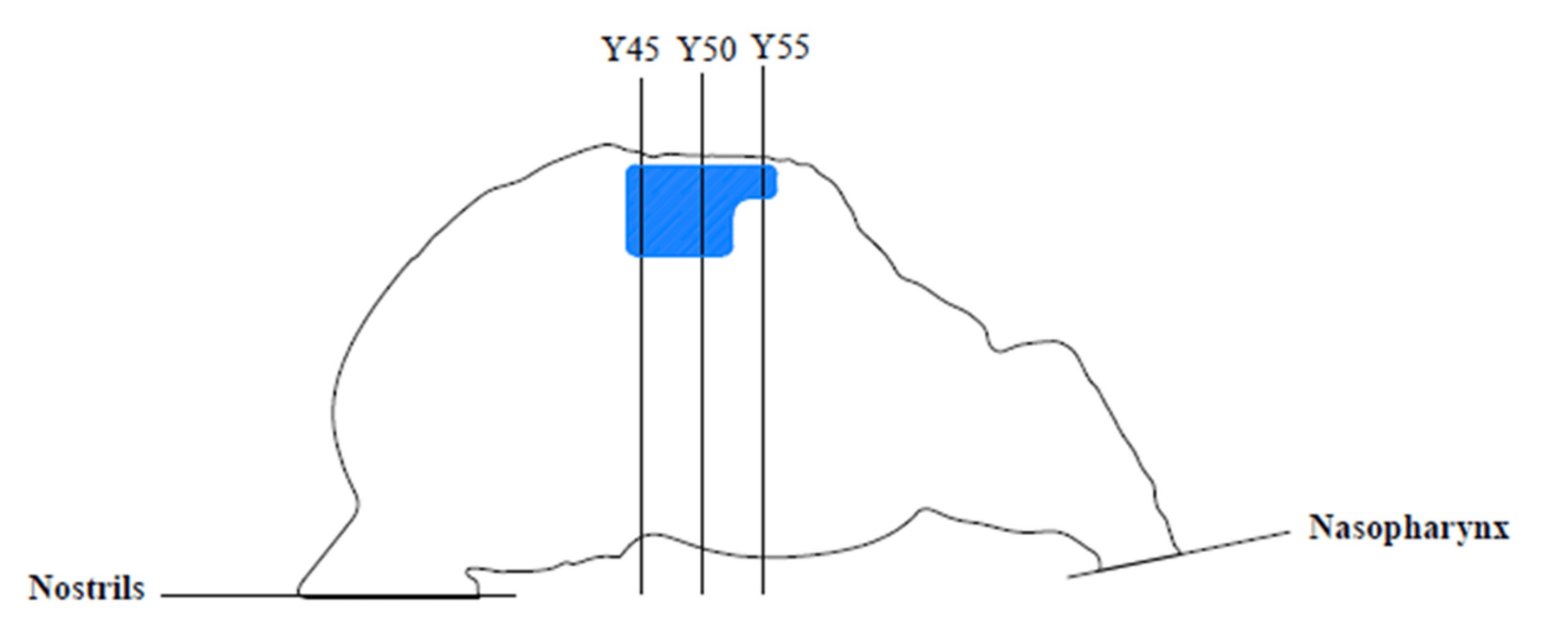
A schematic drawing of the sagittal shape of the main nasal cavity with a schematic representation of planes for displaying results. The planes are named by the percentage of the total length. For example, Y45 is the XZ plane at 45% of the total nasal cavity length from the nostrils. Author's own creation

Several planes were created to plot the characteristics of the flow field. The important planes are the planes of the nostrils and the plane in the nasopharyngeal region. On these planes, mainly the static and total pressure values are displayed as area-weighted averages. Because distance values vary from patient to patient (the size and shape of the nasal cavities are individual), a percentage distance is introduced to present the results of this paper. Coronal planes are created that progress from the nostrils to the nasopharynx. The maximum length of the model from the nostrils to the nasopharynx is subtracted, and planes are produced at 45%, 50%, and 55% of this distance from the nostrils. The results of the velocity and pressure fields are displayed on the coronal planes, schematically shown in Figure 5.

Statistical analyses

Statistical analyses were performed to evaluate the significance of differences observed before and after the intervention. A paired t-test was employed to compare dependent data sets, as this method is appropriate for assessing changes within the same subjects over time. The test accounts for the correlation between paired observations, ensuring accurate evaluation of mean differences. Statistical significance was set at p < 0.05. All analyses were conducted using Matlab R2021a.

## Results

**Figure 6:**
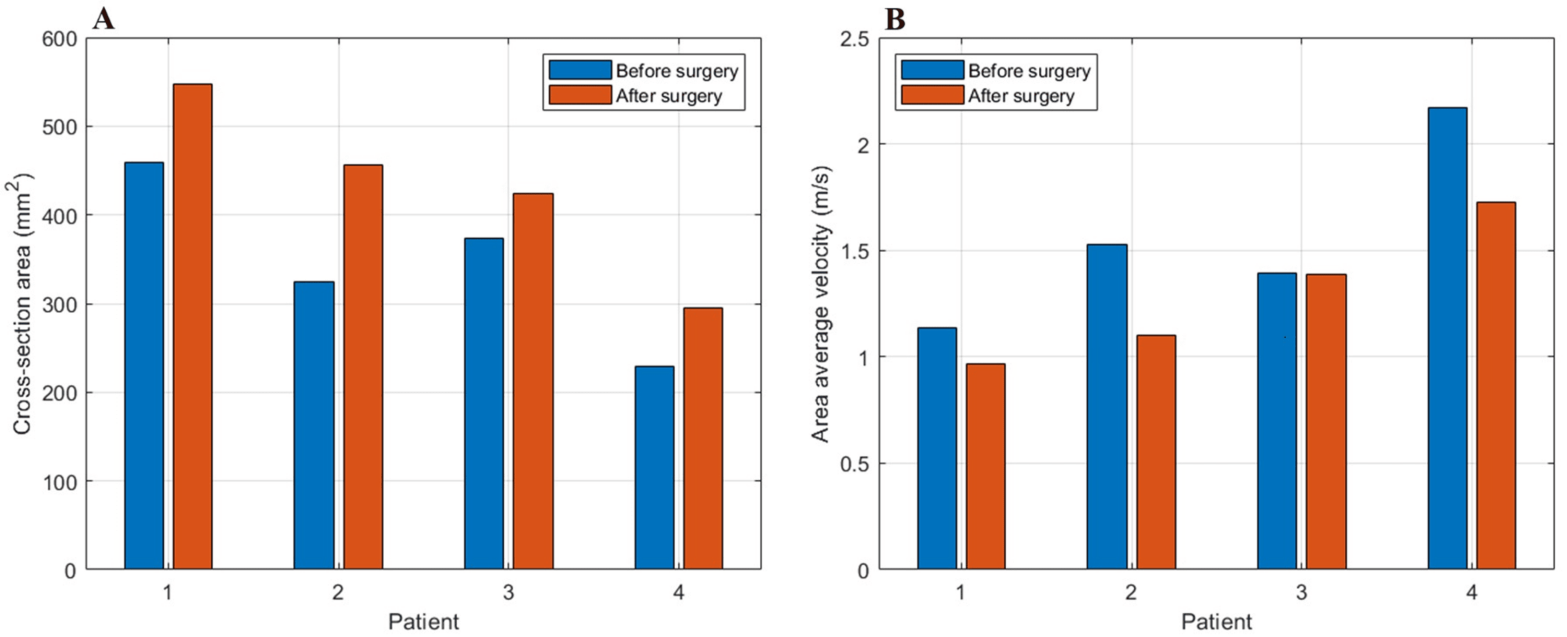
A) Comparison of cross-section enlargement before and after surgery in plane Y55. B) Area average velocity - comparison before and after surgery on plane Y55. Author's own creation

The lateralization of the middle turbinate enlarged the left middle nasal meatus, as well as the passage of the endoscope and surgical equipment. As shown in Figure 6A, the expansion of the middle meatus of the nasal cavity was accompanied by an increase in the surface area of the coronal planes.

**Figure 7:**
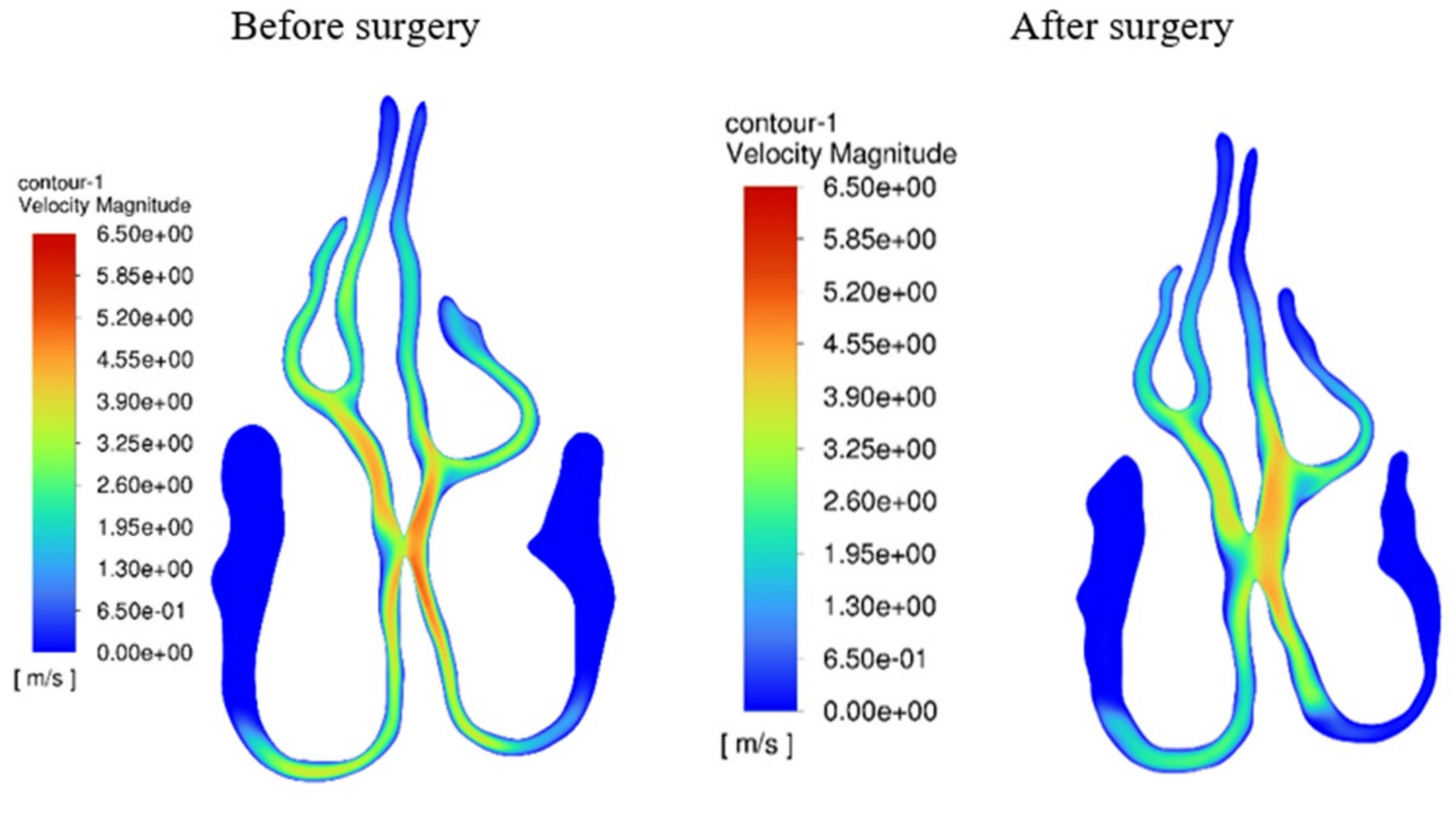
Velocity field of patient 2 in plane 45 - increase in the cross-sectional area of the middle meatus, leading to a decrease in velocity, which represents similar findings to all other models. Author's own creation

The increase in the cross-sectional area was demonstrated by the results of the area average velocity (Figure 6B). We can see that the continuity equation is taking place because the area average velocity must decrease as the area grows. This also refers to the portion of the nasal cavity through which airflow passes. The areas with the least resistance are where airflow occurs. As a result, the middle nasal meatus serves as the passageway for most of the airflow. In consequence, airflow through the peripheral zones is reduced, as can be seen in Figure 7. One of these peripheral zones is the olfactory region. The decrease in velocity between before and after surgery is not statistically significant due to the small number of patients (p-value = 0.091).

The volume flow rate in Table 2 represents the percentage of the volume flow rate in the olfactory region compared to the total flow rate on the corresponding planes. To define the olfactory region, we used the upper 15% of the total vertical height of the nasal cavity cross-section in each coronal plane. This region corresponds approximately to the location of the upper nasal meatus and serves as a proxy for the olfactory cleft. It does not directly represent the olfactory epithelium itself, as this structure cannot be reliably segmented from CT data. Instead, the selected area reflects the surrounding anatomical boundaries and enables consistent comparison of airflow distribution before and after surgery. Volume flow rate was then measured within this region and expressed as a percentage of the total volume flow rate across the entire plane.

**Table 2:** Volume flow rate in the olfactory region on planes Y45 and Y50.

Volume flow rate (%)	Patient 1		Patient 2		Patient 3		Patient 4	
	Before	After	Before	After	Before	After	Before	After
Plane Y45	2.98%	2.54%	1.61%	0.78%	3.36%	0.67%	4.76%	5.42%
Plane Y50	4.40%	3.72%	1.22%	0.19%	4.65%	1.63%	3.29%	4.58%

**Figure 8:**
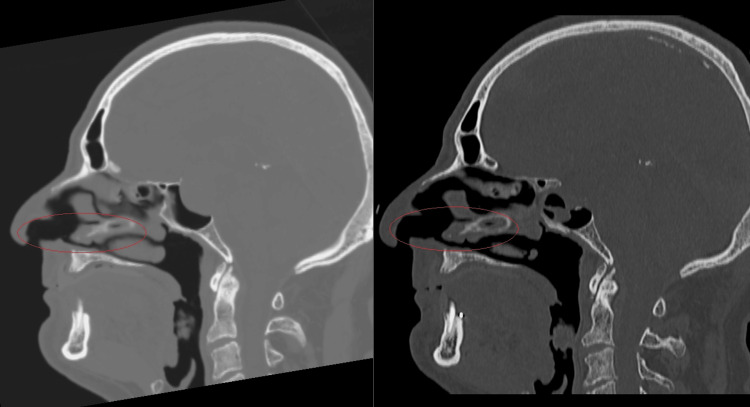
Sagittal planes in patient 1 – on the left before surgery and on the right after surgery. Author's own creation

During the adenoma surgery for patient 1, lateralization of the deviated septum was performed during the approach. This procedure was necessary to access the sphenoid sinus and remove the tumour. Manipulation of the septum results in additional changes in the sinus anatomy that affect the direction of airflow. Important to note are the changes in the geometry of the model in the anterior part of the nasal cavity. Figure 8 shows these changes in the sagittal planes of patient 1 obtained during the CT scan.

**Figure 9:**
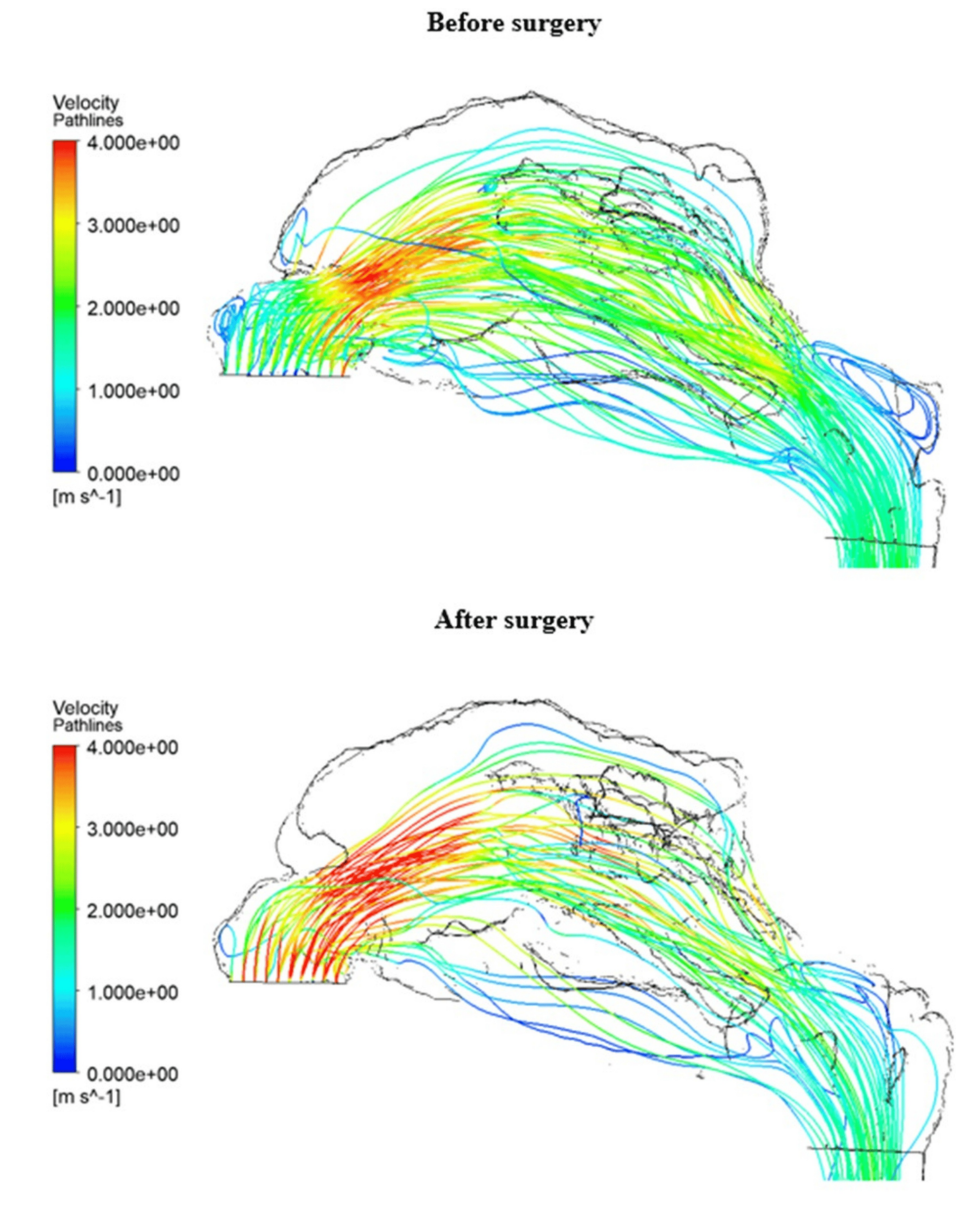
Pathlines in the patient 1 model before and after surgery. Redistribution of air flow to the middle part of the nasal cavity due to enlargement of the region after nasal vestibule. Author's own creation

It is possible to observe an enlargement of the region after the vestibule of the nasal cavity. After surgery, the shape of the enlarged region is slightly sharper, and airflow into the upper nasal meatus encounters greater resistance. At the same time, enlargements can be observed in the lower nasal meatus and in the middle region of the nasal cavity. Figure 9 displays the pathlines. It is possible to compare the change in airflow. After surgery, a minimal amount of air enters the upper nasal meatus as the air flow reverses direction.

**Figure 10:**
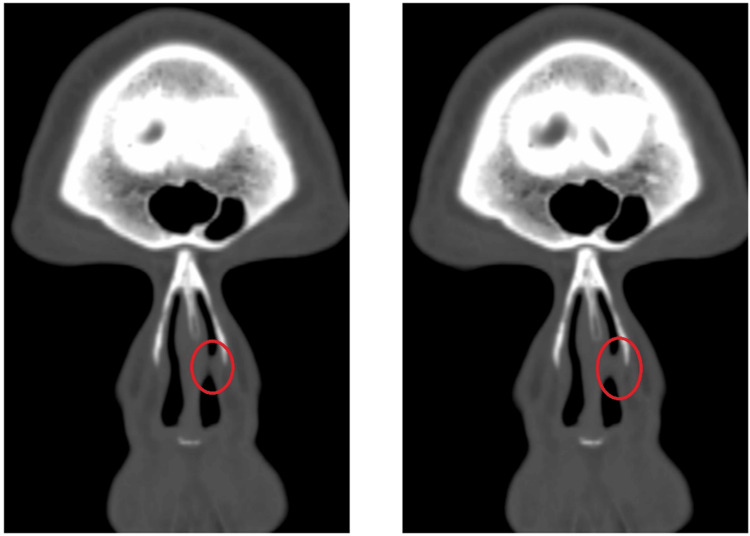
Synechia after surgery in the nasal cavity of patient 4 on two CT slices. Author's own creation

**Figure 11:**
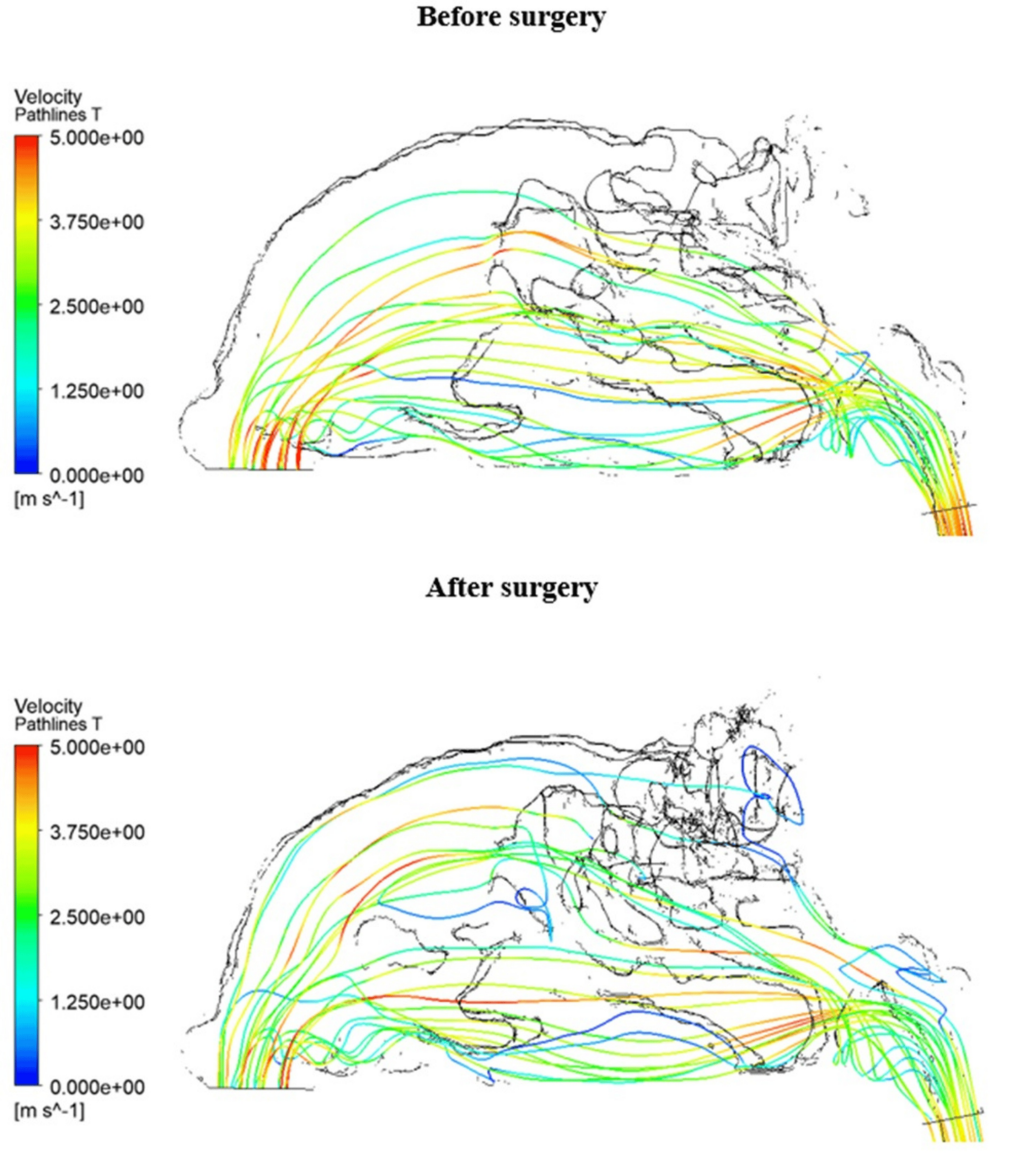
Pathlines in the patient 4 model before and after surgery. Redistribution of airflow to the upper and lower parts of the nasal cavity after surgery due to the obstruction created by the synechia. Author's own creation

The phenomenon of reduced airflow in the olfactory region does not apply to patient 4. In this patient, airflow is divided into the lower and upper meatus due to the operation. This redistribution of flow is caused by the impact of the air flowing into the synechia after entering through the left nostril. The momentum of the flowing medium redirects the airflow to the lower and upper meatus. The synechia after surgery in patient 4 is displayed in Figure 10. Figure 11 shows the difference in airflow in patient 4 before and after surgery. The air redistribution described above in the anterior part of the nasal cavity is observed.

**Figure 12:**
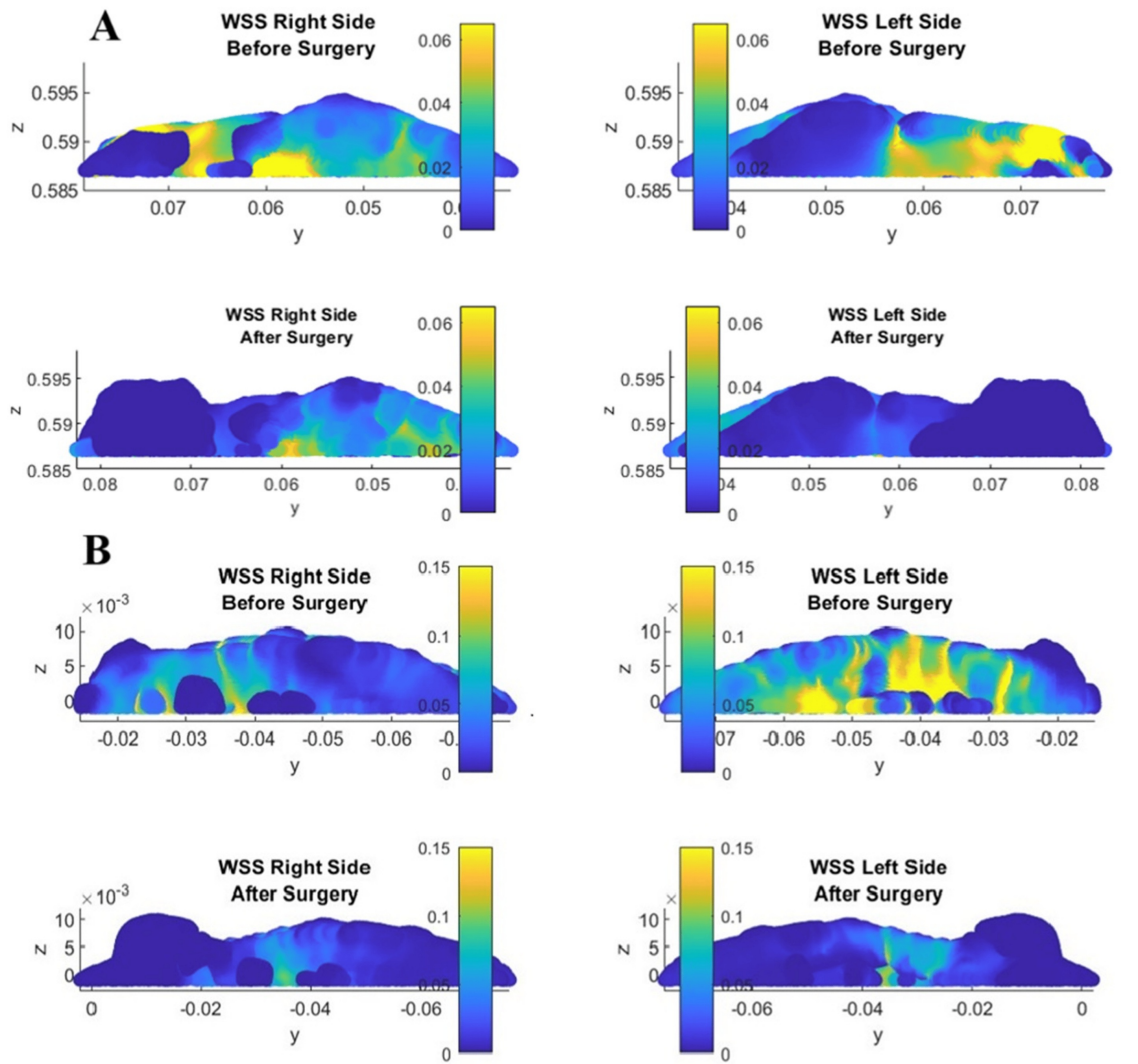
A) Wall shear stress (WSS) in the olfactory region of patient 2 and B) WSS in the olfactory region of patient 3. Author's own creation

The olfactory region in this case can also be considered a peripheral zone. There is a reduction in velocity and flow in the upper nasal passage area, so there may be an alteration in olfactory function. Therefore, another parameter, wall shear stress (WSS), was used to evaluate the effect of the change in the geometry of the nasal cavities on the olfactory function. Figure 12 represents the WSS of two patients (patients 2 and 3) with typical anatomical structures. After the procedure, the WSS decreased, as can be seen in Figure 12. We predict that this could result in a decline in olfactory function, although this theory needs to be verified by additional studies focused on the results of the flow field, WSS, and olfactory measurement.

## Discussion

In pituitary tumor surgery, the middle turbinate must be returned to the correct location after surgery is complete. Exercise of caution during surgery is important to avoid mechanical damage to the mucosa that lines the nasal cavity, because it can lead to a decrease in olfactory functions, as shown in Netuka et al.'s study [26]. Olfactory functions are important in everyday life and can also affect the emotional state of patients (e.g., regarding depression), as Rochet et al. [4] and Croy and Hummel [5] suggest.

The presented results show an increase in the volume of the nasal cavity after passing the endoscope and instruments used during the operation. There is also a decrease in the average volumetric velocity in the various cross sections. Therefore, there is a flow redistribution in all patients, since the volume flow before and after surgery must be the same. In another study [7], CFD was used to investigate the differences following middle turbinate resection. The findings indicated a decrease in resistance and an increase in volume flow rate, which could potentially enhance olfactory functions. In our study, this outcome was observed only in the results of patient 4, where the volume flow rate in the olfactory region increased post-surgery, as shown in Table 2. Figure 11 also illustrates the redistribution of airflow after surgery to the olfactory region. However, the results for the other three patients indicated a decrease in the volume flow rate in the olfactory region. Thus, our findings highlight two key points: the most critical part of the nasal cavity for airflow changes is the anterior part, located behind the nasal vestibule, and an increase in the middle nasal meatus does not appear to enhance olfactory functions, a conclusion that warrants further investigation using additional olfactory measurement methods. Both points need to be verified on a larger sample of patients.

The use of WSS to evaluate the effect of functions of the nasal cavity is also examined in other studies [27,28,29]. The use of WSS as a parameter in this article is based on a similar hypothesis mentioned in [29], where the interaction of the flowing medium with the wall of the nasal cavity may lead to the interaction of odours with olfactory receptors. One of the findings in a study [8] is a decrease in WSS after middle turbinate resection. This could also be connected to the decrease in volume flow rate in the olfactory region.

Due to modifications to the anterior region of the nasal cavity during surgery, two study patients exhibited unanticipated airflow redistribution. The middle meatus and the region in front of the nasal cavity grew larger in patient 1. This enlargement causes airflow to be redirected toward the middle and inferior nasal meatus. Patient 4 experienced synechia in front of the nasal cavity during the healing process. In this scenario, the upper and inferior nasal meati get most of the redistributed airflow. Both irregularities support the idea that even small adjustments in the anterior region of the nasal cavity could significantly affect general airflow redistribution, which can lead to a change in olfactory functions. The study by Hong et al. [14] suggests that total obstruction of the olfactory region and obstruction in the anterior part of the olfactory cleft are connected to a decrease in olfaction. This also supports our hypothesis that if the airflow can’t reach the olfactory region due to changes in the anterior part of the nasal cavity, it could impact olfactory functions.

Study limitations

Some parameters and boundary conditions, such as air temperature, inspiration pressure, and nasal mucosa cycle, may modify nasal airflow. Nasal function can vary without necessarily changing nasal airflow. We also simplified aspects of the CFD calculations by removing secondary nasal sinuses. Future research should focus on the correlation between CFD modifications and changes in olfactory function measured by reliable and valid olfactory tests.

## Conclusions

In this pilot study, we established the technique for modelling nasal airflow in patients before and after resection of the pituitary adenoma. We found a significant increase in the nasal cavity volume after surgery in all four patients, specifically in the middle nasal meatus. It means that most of the nasal airflow is distributed in this area. This change in anatomy leads to a reduction in flow in the specific parts of the nasal cavity, including the olfactory region, and may lead to decreased olfactory functions. Another finding is that even minor changes in anatomy in the anterior part of the nasal cavity (rostrally to the turbinates) can lead to significant and hardly predictable changes in nasal airflow.

Despite the limitations of the present study, the findings can be translated into suggestions for clinical practice. Neurosurgeons should concentrate on repositioning the middle turbinate and septum at the end of surgery to prevent changes in nasal airflow, especially in the olfactory area. In addition, special attention should be paid to the preservation of the anterior part of the nasal cavity. Our conclusions need to be verified by further studies using a clinical olfactory examination.
